# Non-Linear Self-Heating in Organic Transistors Reaching High Power Densities

**DOI:** 10.1038/s41598-018-27689-3

**Published:** 2018-06-28

**Authors:** Markus P. Klinger, Axel Fischer, Hans Kleemann, Karl Leo

**Affiliations:** 1 Dresden Integrated Center for Applied Physics and Photonic Materials (IAPP), Technische Universität Dresden, Nöthnitzer Str. 61, 01187 Dresden, Germany; 2 Center for Advancing Electronics Dresden (cfead), Technische Universität Dresden, Würzburger Str. 43, 01187 Dresden, Germany

## Abstract

The improvement of the performance of organic thin-film transistors is driven by novel materials and improved device engineering. Key developments are a continuous increase of the charge carrier mobility, a scale-down of transistor dimensions, and the reduction of contact resistance. Furthermore, new transistor designs such as vertical devices are introduced to benefit from drastically reduced channel length while keeping the effort for structuring moderate. Here, we show that a strong electrothermal feedback occurs in organic transistors, ultimately leading to output characteristics with regions of S-shaped negative differential resistance. For that purpose, we use an organic permeable-base transistor (OPBT) with outstanding current densities, where a strong and reproducible, non-linear electrothermal feedback is revealed. We derive an analytical description of the temperature dependent current-voltage behavior and offer a rapid investigation method for material systems, where a temperature-activated conductivity can be observed.

## Introduction

The vision of future flexible electronic circuits based on organic semiconductors has triggered an impressive scientific development, aiming for an increased charge carrier mobility in these semiconductor materials^1^. Along with improved processing techniques, charge carrier mobilities of *µ* > 10 cm^2^ V^−1^s^−1^ ^2–4^ have been demonstrated in organic thin-film transistors. Owing to this development, organic electronic devices become increasingly interesting for high-frequency applications such as short range wire-less communication^5^. The highest cutoff frequency of an organic transistor is *f*_T_ = 40 MHz so far^6^, but optimized devices and geometries should allow for an operation beyond 100 MHz as already demonstrated by using inorganic materials^7–9^. For devices based on inorganic semiconductors, heat dissipation is a major concern since high charge carrier mobilities allow for very high current densities^10^. Two types of behavior are possible: On the one hand, field-effect transistors where the charge carrier mobility and the electrical conductivity decrease with temperature, e.g. due to increased phonon scattering, reveal either a more pronounced saturation regime or even an N-shaped negative differential resistance (N-NDR) upon self-heating. Mostly, field-effect transistors show this kind of behavior in the saturation regime of the output characteristics^11–15^. On the other hand, bipolar transistors can show S-shaped negative differential resistance, typically seen in the Gummel plot^16–18^. In these devices, the transmission current increases with temperature due to increasing charge carrier densities. Of course, self-heating effects are also reported for transistors made from many other materials, e.g. amorphous silicon, low temperature polysilicon, oxide semiconductors as well as 2D semiconductors^19–23^. In contrast, only a few publications consider self-heating for organic transistors^24–26^. Although typical substrates (glass, polymer) have a very low thermal conductivity promoting Joule self-heating, the power dissipation of most of the organic transistors does not result in pronounced non-linear self-heating effects at room temperature. In organic semiconductors, charge carrier transport is described by hopping of charges between localized states rather than band-like transport. Since the hopping process becomes more effective with increasing temperature, usually a positive thermal activation energy of charge carrier mobility and conductivity is observed^27^. As demonstrated, such a positive thermal activation of charge carrier conductivity leads to a self-accelerated increase of device temperature and current, eventually leading to S-NDR and thermal switching^28^. This kind of behavior, however, is only been demonstrated for two-terminal crossbar structures, such as n-i-n electron-only devices or organic light-emitting diodes, as these devices easily heat up during operation. Here, we demonstrate a pronounced electrothermal feedback at room temperature for organic transistors. For that purpose, we use an organic permeable-base transistor (OPBT) for which we recently reached current densities of 1 kA cm^−2^ ^8^. A strong, reproducible, non-linear electrothermal feedback is revealed. We then derive an analytical description of the temperature dependent current-voltage behavior, and check whether independently measured activation energies of the electrical conductivity can explain the experiment. Finally, we discuss the prospects which arise due to the strongly temperature activated conductivity.

## Results

### Transistor Setup

**Figure 1:**
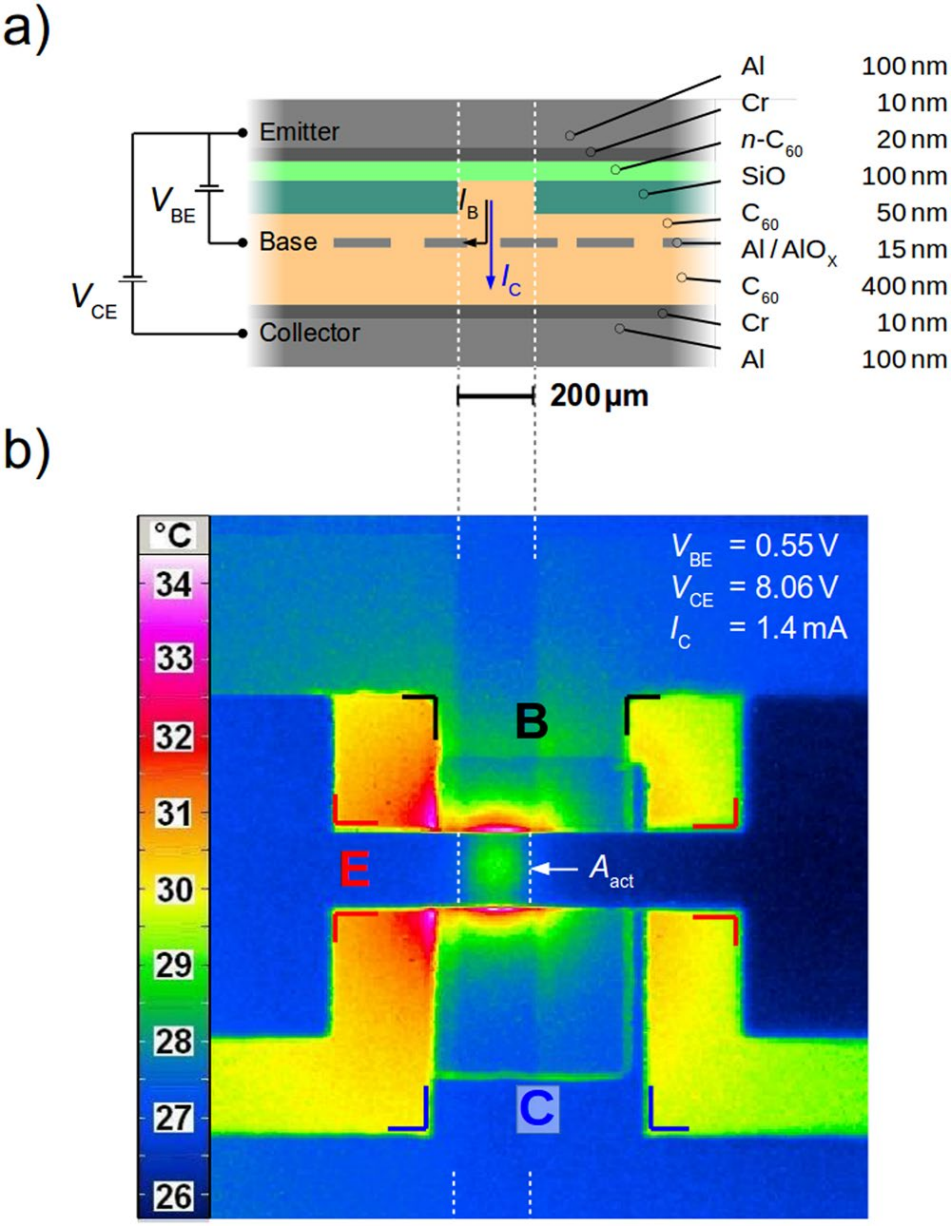
(**a**) Schematic device cross-section and electrical circuit in common-emitter configuration. Materials: aluminum (Al), chrome (Cr), n-doped C_60_ (n-C_60_), intrinsic (undoped) C_60_ (i-C_60_), native aluminum-oxide (AlO_X_). Arrows indicate the electron flow. Additional insulating layers (SiO) are inserted for defining and down-scaling the active area. **(b)** The thermal imaging during the S-NDR measurement confirms the increased temperature in the active area *A*_act_ of the OPBT.

**Figure 2:**
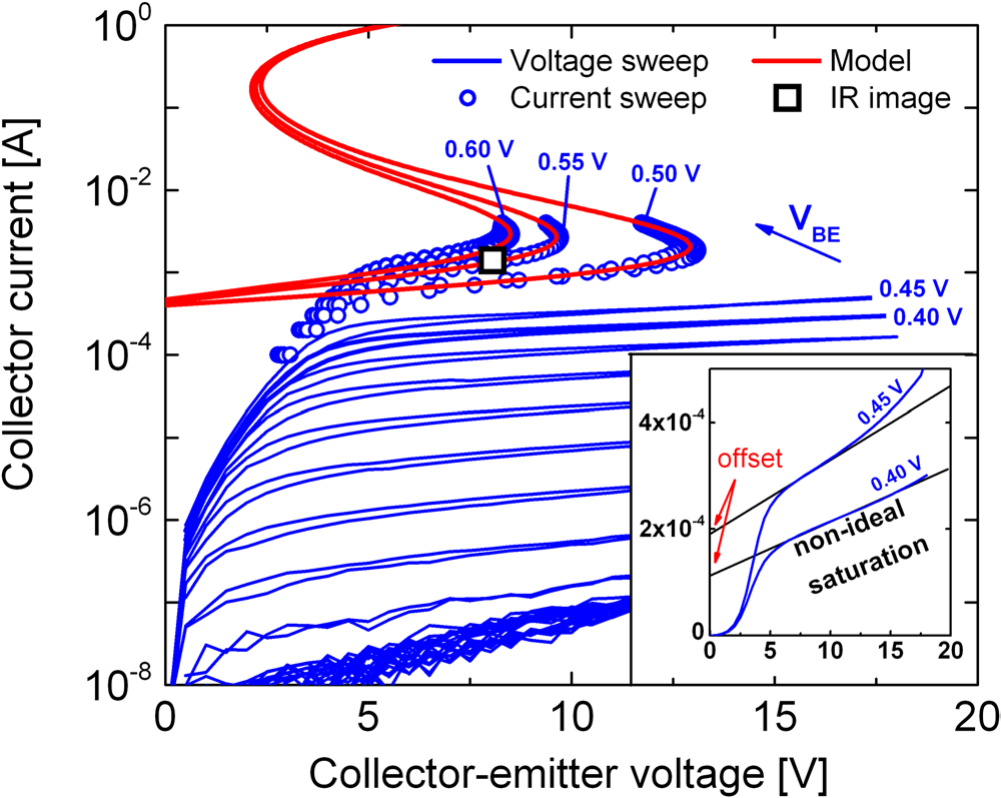
Output characteristic of an OPBT revealing S-NDR behavior. At low base-emitter voltages, a voltage sweep (blue lines) is used. In order to stabilize the NDR at base-emitter voltages starting from *V*_BE_ = 0.5 V, a current controlled measurement (blue circles) is used. All curves are measured with forward and backward sweep, demonstrating the repeatability of the self-heating effect. The model (red lines), assuming an Arrhenius-like temperature activation of the conductivity, leads to a reasonable agreement with the experimental data. The black squared point indicates where the thermal image is taken, cf. Fig. 1(b). Inset: The OPBT shows a non-ideal saturation behavior. It can be described by a linear curve with an additional off-set.

For our study, we use an OPBT as shown in Fig. 1(a). The device consists of a simple sandwiched architecture, using three electrodes which are separated by two intrinsic C_60_ layers. The vertical current between the two outer electrodes, emitter and collector, can be controlled by a permeable base electrode which allows for vertical charge transport through nano-size openings^29^, [143 et sqq.]. Due to air exposure of 15 min after processing, the 15 nm thin middle Al electrode creates a native surface oxide which is insulating and supports the charge carrier transmission through the pinholes formed during a post heat treatment^30^. Details of the working mechanism including this charge carrier transmission through the base have been simulated and investigated^31^. The best performance is reached by using contact-doping at the top emitter electrode, strongly reducing the contact resistance, so that the on-state of these devices is mainly limited by the charge transport through the intrinsic layers^8,30^. We use a combination of chromium and aluminum for the outer electrodes which yields low resistivity and prevents the aluminum from oxidation, there. Further improvements can be achieved by inserting insulating layers of thermally evaporated SiO in order to reduce the active area of these devices^32^. According to these optimizations, OPBTs reach current densities of 1 kA cm^−2^ in pulsed operation, corresponding to a significant power dissipation of about 3.5 W on an area of 200 µm × 200 µm. To clearly work out self-heating effects in OPBTs, we concentrate on the saturation regime of the output characteristics. Please note that we generally observe an additional linear contribution to the current-voltage behavior in that regime, as the base electrode does not fully screen the emitter and collector potential (see inset of Fig. 2). The bottom intrinsic thickness is increased from 100 nm to 400 nm, cf^32^. According to a field stability of 1 MV/cm of the base-collector diode, a voltage stability of about 40 V can be achieved^32^ and higher input powers are reached by applying higher voltages keeping the current level as low as possible. Furthermore, the adapted design increases the on-state resistance of the transistor and thus decreases the influence of a residual external series resistance of the electrodes. Still, the OPBT easily reaches the self-heating regime at voltages of about 10 V as discussed in the following.

Figure 1(b) shows a thermal image of the OPBT operating at a current of *I*_C_ = 1.4 mA and a voltage of *V*_CE_ = 8.06 V reached at a *V*_BE_ = 0.55 V. In horizontal direction the emitter electrode and in vertical direction a trench of missing insulating material, both having a width of 200 µm, define the final active area *A*_act_. The base electrode, coming from the top, and the collector electrode, coming from the bottom, have a width of 600 µm in order to ensure that they are present in the desired active area. We can not perform an exact calibration of the temperature as each layer of our structure has a different emissivity of the thermal radiation, which is also why investigated edge effects occur^33^. Still, the thermal resistance can be estimated to be in the range of 1000 K W^−1^ in accordance with other measurements of a similar device geometry and same substrate material^28,33^.

### Experimental evidence of S-NDR

The effect of self-heating can be seen in Fig. 2 where output characteristics of the OPBT are shown for various base-emitter voltages. Here, two measurement approaches are used. Starting with low *V*_BE_ from −0.5 V to 0.45 V, a voltage-controlled sweep of *V*_CE_ is performed. In this range, the achieved power densities are too low to reach substantial self-heating, but a first influence can be observed for a *V*_BE_ of 0.45 V, cf. inset in Fig. 2. More pronounced self-heating occurs when a base-emitter voltage of 0.5 V, 0.55 V, or 0.6 V is applied. For example, we highlight the point (black square) at which the thermal image is taken in Fig. 1(b) and can confirm that at this power dissipation, self-heating gets significant. Now, a current controlled measurement is used which is necessary to stabilize the current-voltage curves when the device enters the S-NDR regime. Otherwise, a thermal runaway would start which ultimately destroys the device. Using that method, we are able to prove the existence of S-NDR in organic transistors as there is a voltage turnover, which occurs at a power of *I*_C_ × *V*_CE_ ≈ 25 mW, so at a different voltage for each current. The effect itself is repeatable as we can reproduce the current-voltage curve in forward and backward direction of the sweep and further we can change the behavior by applying different base-emitter voltages. We find that the turnover point shifts to lower currents but higher collector-emitter voltages for a lower base-emitter voltage, which is another proof that the voltage turnover depends on the power dissipation.

### Modeling

In order to quantitatively describe the thermal feedback, a model accounting for the temperature dependence of the electrical conductivity needs to be derived. As mentioned above, in almost all organic semiconductors, charge carrier transport is described by a hopping of either electrons or holes between localized states in Gaussian density of the states (DOS). Since these jumps occur upon thermal activation, the dependence of the charge carrier mobility *µ* on temperature *T* obeys

1 $${\mu }(T)\propto \exp (-\frac{{\rm{const}}{\rm{.}}}{{T}^{m}})$$

where *m* varies from the analytical solution 2 (Gaussian DOS) to 1^27,34^. At high charge carrier concentration (*m* = 1), the mobility has an Arrhenius-like temperature dependence which is often used for describing experimental data and can also be used for a local approximation when *m* = 2. In order to phenomenologically model the temperature activated conductivity *σ*(*T*) = *σ*_0_*F*(*T*) we use an Arrhenius-like temperature activation factor

2 $$F(T)=\exp [-\frac{{E}_{{\rm{act}}}}{{k}_{B}T}(\frac{{\rm{1}}}{T}-\frac{{\rm{1}}}{{T}_{a}})]$$

with *m* = 1, *T*_a_ being the ambient temperature and *E*_act_ being an effective activation energy describing the entire device behavior. Such an approach is used in ref.^28^ to successfully describe the electrothermal feedback in a simple two-terminal device. The introduced factor *F*(*T*) in Eq. 2 can be any monotonically increasing function. However, the Arrhenius-like law includes the advantage to yield an activation energy as fitting parameter which can be used for comparison with other systems and devices.

A transistor, however, displays a current-voltage behavior depending on biasing conditions, namely the linear and the saturation regime. Similar to other transistors, OPBTs also show these two distinct regions in the current-voltage curve (cf. inset Fig. 2). In the linear regime, the conductivity is so high that the transistor is easily restricted by space charge limited currents in the intrinsic C_60_ layers^8,31^. In the saturation regime, short channel effects hinder having an ideal saturation, which can be described by a linear current-voltage relation with a current offset (see inset Fig. 2).

In order to model the current-voltage behavior of an organic transistor in this non-ideal saturation regime, we extend the power law model in ref.^29^ by a temperature-activated offset current *I*_off_ and employ

3 $$I(V,T)=\mathop{\underbrace{{I}_{{\rm{r}}{\rm{e}}{\rm{f}}}{(\frac{V}{{V}_{{\rm{r}}{\rm{e}}{\rm{f}}}})}^{a}{F}_{1}(T)}+\mathop{\underbrace{{I}_{{\rm{o}}{\rm{f}}{\rm{f}}}{F}_{2}(T)}}\limits_{{\rm{o}}{\rm{f}}{\rm{f}}{\rm{s}}{\rm{e}}{\rm{t}}}}\limits_{{\rm{p}}{\rm{o}}{\rm{w}}{\rm{e}}{\rm{r}}\,{\rm{l}}{\rm{a}}{\rm{w}}}$$

where each term has an own temperature activation factor *F*_1_(*T*) and *F*_2_(*T*). In Eq. 3, we introduce three parameters describing the isothermal current-voltage curve at ambient temperature: an exponent *α*, a reference point of the curve with the current *I*_ref_ and the voltage *V*_ref_.

In order to include electrothermal feedback, the power dissipation has to equal the heat *Q*_1_ = (*T* − *T*_a_)/*Θ*_th_ which can be transported away at a certain global thermal resistance *Θ*_th_. In our homogenous model, the temperature increase represents a kind of average device temperature. This approach can be used because the width of the active area is small, especially in comparison to the thickness of the substrate, which attenuates temperature gradients within the device by lateral heat flow.

The conservation of energy results in

4 $${V}^{\alpha +{\rm{1}}}+\frac{{I}_{{\rm{off}}}{F}_{{\rm{2}}}(T){V}_{{\rm{ref}}}^{\alpha }}{{I}_{{\rm{ref}}}{F}_{{\rm{1}}}(T)}V-\frac{{V}_{{\rm{ref}}}^{\alpha }(T-{T}_{a})}{{I}_{{\rm{ref}}}{\Theta }_{th}{F}_{{\rm{1}}}(T)}={\rm{0}}$$

having the polynomial structure *V*^*α*+1^ + *aV* + *b* = 0 where *a* and *b* are constants.

**Figure 3:**
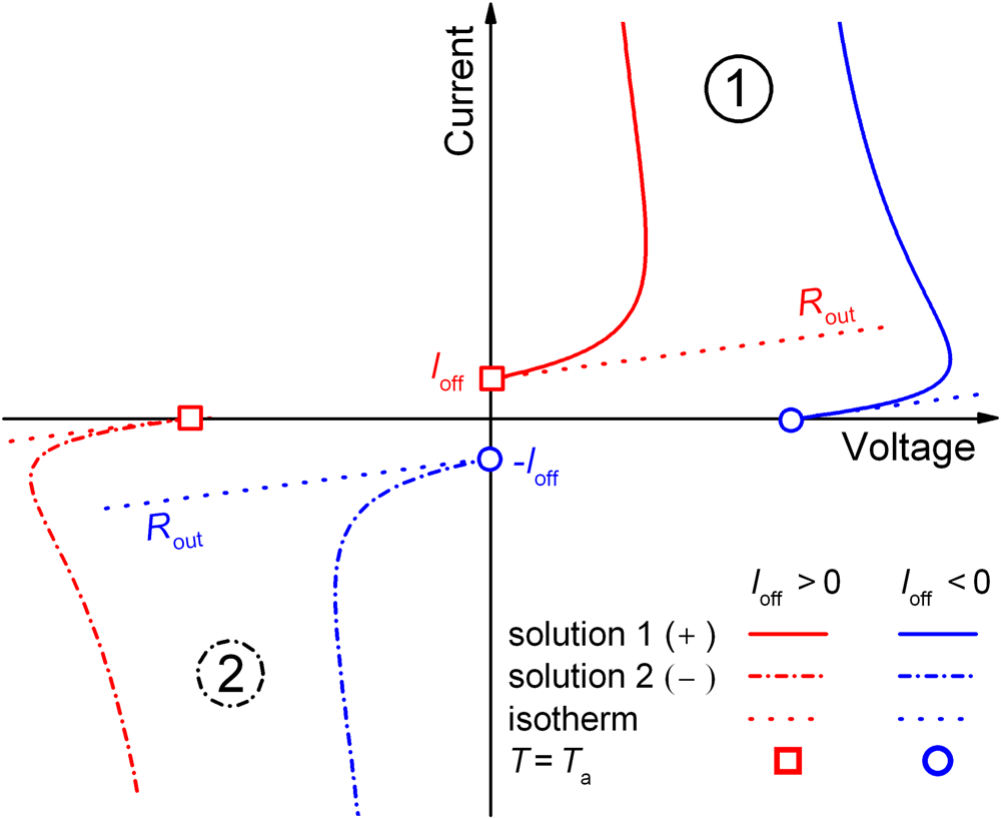
Visualization of the analytical solutions of Eq. 5. The two solutions 1 (+, solid) and 2 (−, dashed) of the red curve (*I*_off_ > 0) have a point symmetry to the two solutions of the blue curve (*I*_off_ < 0). The two curves in the first quadrant represent either self-heating of a transistor in the non-ideal saturation regime of the output characteristic (red) or self-heating of a rectifying diode in forward direction (blue). At the points where no Joule self-heating occurs (*T* = *T*_a_, *I* = 0 A or *V* = 0 V), the solutions coincide with the isothermal current-voltage relation.

To achieve an analytical solution, we simplify the above equation in the following manner: The exponent *α* is set to 1, as the non-ideal saturation regime can be described by a linear law having the differential output resistance *R*_out_ = *V*_ref_/*I*_ref_. Further, we assume that the power law part and the constant current offset have the same temperature activation factor *F*_1_(*T*) = *F*_2_(*T*) = *F*(*T*). These conditions result in a quadratic equation which can be solved analytically. We obtain the two solutions

5 $${V}_{1,2}=-\frac{{I}_{{\rm{off}}}{R}_{{\rm{out}}}}{{\rm{2}}}\pm \sqrt{{(\frac{{I}_{{\rm{off}}}{R}_{{\rm{out}}}}{{\rm{2}}})}^{{\rm{2}}}+\frac{{R}_{{\rm{out}}}(T-{T}_{a})}{{\Theta }_{{\rm{th}}}F(T)}}$$

which are visualized conceptually in Fig. 3. Solution 1 is in the first quadrant and solution 2 is in the third quadrant. Both solutions are related to a positive increase of the temperature difference *T *− *T*_a_. Mathematically possible solutions in the second and fourth quadrant are omitted as they would be related to a negative power dissipation and a fictive cooling of the device.

For the red curve (*I*_off_ > 0), the first solution is the one which can be used to describe self-heating of a transistor in the non-ideal saturation regime. However, the second solution is an independent solution which can further be visualized by making a point symmetry transformation (*I* → −*I*, *V* → −*V*) related to blue lines in Fig. 3. These transformed curves can also be gained if Eq. 5 is used with a negative offset current (*I*_off_ < 0). Thus, solution 1 of the red curve belongs to solution 2 of the blue curve and vice versa. Now, the blue dashed isothermal curve comes out of the *x* axis in the first quadrant and this solution corresponds to an electronic device which has a linear current-voltage increase after a certain turn-on voltage. For example, this would suit to describe a rectifying diode in forward direction^35^.

For completeness, the case of an ideal saturation with *R*_out_ → ∞ is discussed. In contrast to Eq. 5, the power law in Eq. 3 is here excluded and only the constant offset results in the solution

6 $$V=\frac{T-{T}_{a}}{{\Theta }_{{\rm{th}}}{I}_{{\rm{off}}}F(T)}.$$

### Fitting procedure

In a next step, we fit the data of Fig. 2 using Eq. 5. For pre-defined temperature values *T* ≥ *T*_a_, the temperature activation factors of the electrical conductivity *F*(*T*) based on Eq. 2 and the voltages *V*_1_(*T*) are calculated. Equation 3 then yields the currents *I*_1_(*T*). Next, voltage-current pairs for each temperature *T* are used to construct the self-consistent current-voltage curve upon self-heating. A series resistance *R*_ser_ can finally be included to correct the voltage *V*(*T*) by adding *I*_1_ × *R*_ser_.

**Figure 4:**
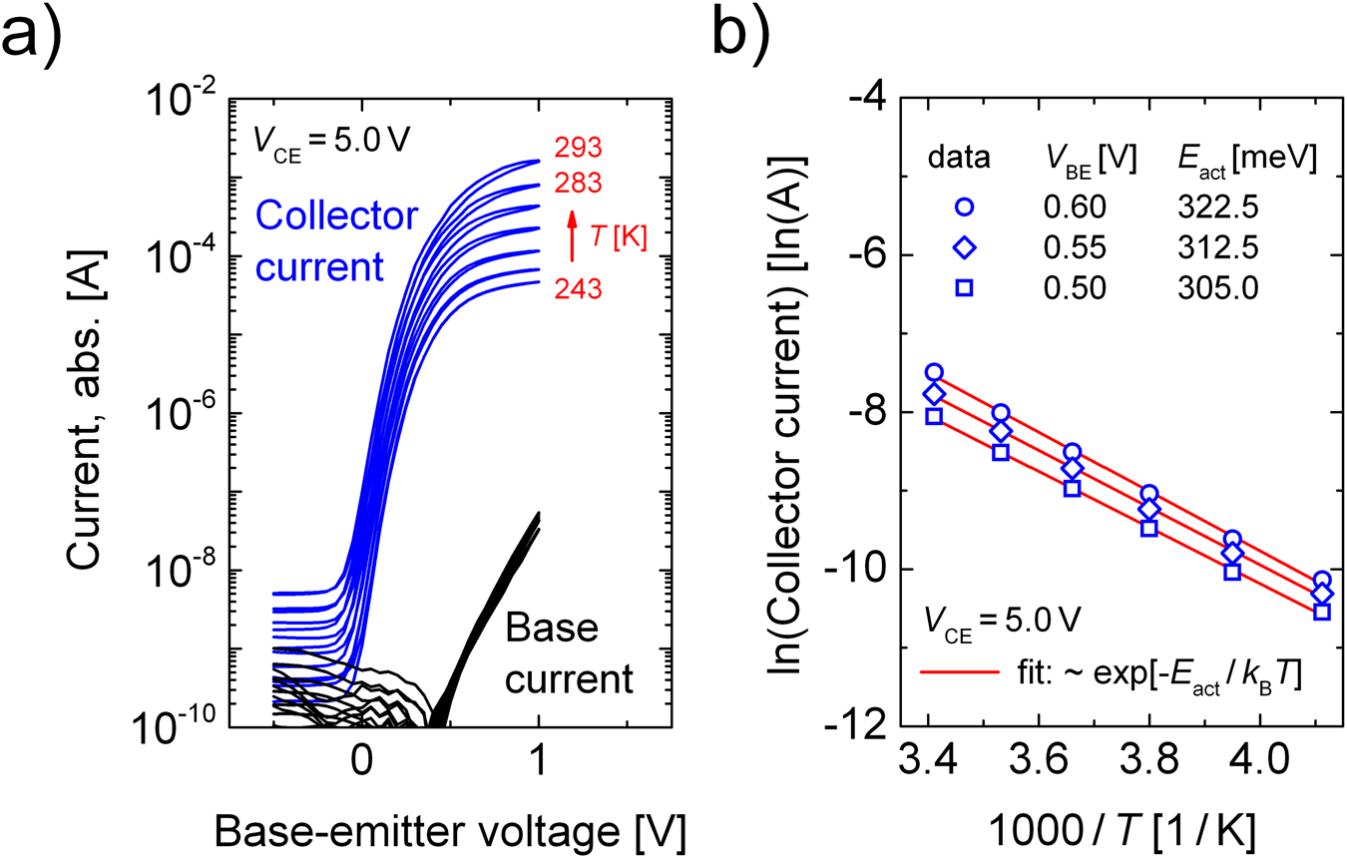
Measurement of the activation energy *E*_act_. **(a)** Temperature dependent transfer curve for an operation voltage *V*_CE_ of 5.0 V. **(b)** Extraction of the activation energy from (**a**) at base-emitter voltages as modeled in Fig. 2.

The analytical approach introduced, cf. Eq. 5, now serves to fit the experimentally found S-shaped NDR behavior in Fig. 2(a) at *V*_BE_ = 0.5 V, 0.55 V, and 0.6 V. In Table 1, all fit parameters are summarized. For *V*_CE_ > 5 V, a non-ideal saturation can be seen for all measured curves and we concentrate on matching this range with our model. The thermal resistance *Θ*_th_ is constantly set to 1090 K W^−1^ for all base-emitter voltages in order to see non-linear self-heating effects exactly in that range where they can also be seen by the experiment. The chosen value is in close proximity to values obtained for devices of similar geometry on glass substrates^28,33^. The offset current *I*_off_, related to the current level of the non-ideal saturation regime, slightly increases with *V*_BE_ from 0.4 mA to 0.47 mA. The output resistance *R*_out_ describing the slope of the isothermal, non-ideal saturation regime is on the range of some 10 kΩ and adjusted to match the curvature of the measured curve. We use a series resistance, attributed to the electrode layout of 5 Ω which is similar to common values of this electrode configuration and mainly influences the second upper turning point (cf. refs^28,33^). The most important parameter function *E*_act_ relevant to achieve a regime of negative differential resistance, is not fitted but is directly taken from independent isothermal measurements (cf. Fig. 4). Thus, the main fitting parameters are the current offset *I*_off_ and the output resistance *R*_out_, both describing the isothermal current-voltage relation at *T*_a_ and the thermal resistance *Θ*_th_.

**Table 1:** List of parameters found to model the non-ideal saturation regime of the output characteristics in Fig. 2.

*V*_BE_ [V]	*α*	*R*_ser_ [Ω]	*Θ*_th_ [KW^−1^]	*E*_act_ [meV]	*I*_off_ [mA]	*R*_out_ [kΩ]
0.50	1	5	1090	305.0	0.40	45.0
0.55	1	5	1090	312.5	0.44	21.0
0.60	1	5	1090	322.5	0.47	16.5

Parameters which are not changed by *V*_BE_ are kept constant.

### Activation energies of conductivity

The activation energies of the OPBT are measured in a cryostat by taking the transfer curves at *V*_CE_ = 5 V, a range in which self-heating effects are less pronounced, cf. Fig. 2, so that we assume each curve to be isothermal. The data can be seen in Fig. 4(a) for temperatures from 243 K to 293 K in 10 K steps.

Higher temperatures are avoided to restrict the maximal achieved power dissipation. All curves have a ratio between the on- and the off-state in the range of 5 to 6 orders of magnitude and shift upwards with temperature, demonstrating that the electrical conductivity of these devices can indeed be increased by Joule self-heating. However, we find that the base current shows no change with temperature at all which we attribute to the fact that the emitter-to-base leakage current is mainly limited by tunnel processes through the thin native AlO_X_ of the base electrode. As a consequence, the current gain of the OPBT constantly increases with temperature by about 4 to 5 orders of magnitude and thus gets comparable to what is achieved for lateral field-effect transistors where more well-defined gate dielectrics are used^36^.

For the base-emitter voltages *V*_BE_ used to model the data in Fig. 2, the activation energy is determined in Fig. 4(b). The measured data obey an Arrhenius-like law following *j* ~ exp[−*E*_act_/*k*_B_*T*] and an OPBT activation energy in the range of 300 to 330 meV is found varying slightly with the applied *V*_BE_. Therefore, it is shown that the S-NDR in our measurement can solely be explained by an Arrhenius law similar to the thermal activation of the electrical OPBT conductivity.

The values of the OPBT activation energy found are in the same range as observed for crossbar structures with similar sample setups^28^. Furthermore, in agreement to drift-diffusion simulations on OPBTs^31^ and intrinsic layers^37^, we obtain a pronounced thickness dependence on the activation energy parameter.

### Comparison with numerical solution

Up to now, the analytical solution is used to model S-NDR behavior based on a positive activation energy. However, our solution can also be applied for the opposite case. For a negative activation energy of the electrical conductivity, the current decreases with temperature and such characteristics are often found in inorganic electronics where charge carrier mobility decreases with temperature^35^. As a consequence, an N-NDR behavior is found which has the drawback that the performance is reduced upon self-heating, but also has the advantage that at the same time thermal runaway is automatically suppressed. To test the generality of our analytical solution and to provide a tool useful for circuit integration, we provide a SPICE model describing the self-heating in a field-effect transistor. In the linear regime (|*V*_GS_ − *V*_th_| > |*V*_DS_|), it is described by

7 $${I}_{D}\propto {\mu }_{0}F(T)\cdot [({V}_{{\rm{G}}{\rm{S}}}-{V}_{{\rm{t}}{\rm{h}}}){V}_{{\rm{D}}{\rm{S}}}-\frac{{V}_{{\rm{D}}{\rm{S}}}^{2}}{2}]\cdot (1+\lambda {V}_{DS})$$

and in the non-ideal saturation regime (|*V*_DS_| > |*V*_GS_ − *V*_th_| > 0) by

8 $${I}_{D}\propto {\mu }_{0}F(T)\cdot \frac{{({V}_{{\rm{G}}{\rm{S}}}-{V}_{{\rm{t}}{\rm{h}}})}^{2}}{2}\cdot (1+\lambda {V}_{DS})$$

where *µ*_0_ is a constant mobility, and *F*(*T*) is the temperature activation factor (s. Eq. 2). The factor (1 + *λV*_DS_) is taken from the Shichman-Hodges model to include a non-ideal behavior^38^, leading to a finite output resistance in the non-ideal saturation regime.

**Figure 5:**
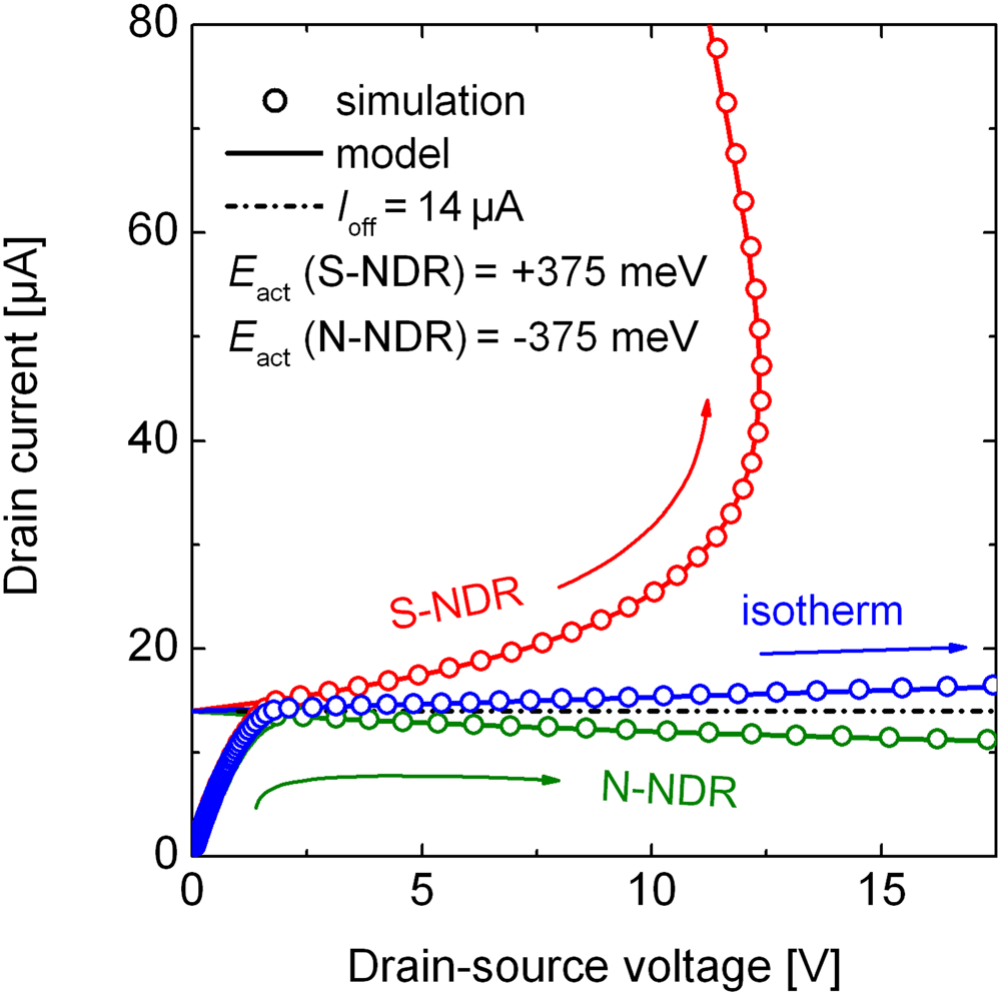
Comparison of the analytical solution with simulation results. The data points of the transistor model are identical to the model for the same set of parameters. Both cases, S-NDR at positive activation energies, and N-NDR at negative activation energies are reproduced.

In Fig. 5, the three scenarios can be seen: S-NDR (+375 meV), an effective isothermal curve (0 meV) and N-NDR (−375 meV).

We use arbitrary parameters and based on the simulation parameters, the offset current is adjusted to be 14 μA, and the output resistance equals 7.5 MΩ. The thermal resistance is set to 40000 K W^−1^. The analytical solution is identical to the simulation in the non-ideal saturation regime if exactly the above named parameters are used. Both, S-NDR and N-NDR, are reproduced. Thus, our analytical solution can also be used to calculate the reduction of current flow, e.g. as seen in MoS_2_ transistors^23^, to check whether the behavior can solely be explained by a temperature dependent conductivity. The unique advantage of the analytical solution is of course that it allows for a direct access to the characteristic of the problem. For example, it can be seen by Eq. 5 that the strength of the non-linear electrothermal feedback solely depends on temperature activation factor and thus on *E*_act_/*k*_B_*T*_a_. Assuming *E*_act_ is constant, the electrothermal feedback gets more pronounced at low ambient temperatures *T*_a_, leading to sharper voltage turnovers and a thermal runaway which starts already at lower self-heating induced temperatures rises.

### Discussion

It is very likely that thermal switching and negative differential resistance due to self-heating will also soon be shown and confirmed for state-of-the-art organic thin-film transistors at room temperature. The continuous improvement in mobility and down-scaling of organic thin-film transistors will give rise to an increased importance of heat management for organic transistors. At very low ambient temperatures (<50 K), Nikiforov *et al*. already observed indications for thermal switching in organic transistors^26^. These findings can be well explained by our model as the electrothermal feedback get stronger at low temperatures. Additionally, our work reveals a purely thermally induced non-linear self-heating effects at room temperature. Interestingly, Matt *et al*. found switching effects in field-effect transistors at high voltages and currents (100 V, 1 mA)^39^. At this time, the origin has not unambiguously been figured out, but based on our findings, thermal switching as reason is very likely.

Power densities (>100 W cm^−2^) should be also achievable by lateral field-effect transistors and temperature rises due to self-heating are already discussed in literature^24–26^. The importance becomes even more clear considering that our transistors are built on glass substrates and even less power densities would be necessary to see similar effects on flexible substrate which have a lower thermal conductivity.

In general, electrothermal feedback is very pronounced in organic materials due to the high activation energies of the electrical conductivity. Here, we like to point out that this is also a chance for organic semiconductor devices. Even small temperature changes can significantly change the charge carrier mobility as well as the conductivities of these materials, leading to an increasing performance during Joule self-heating. Thus, operation at a controlled level of self-heating can also be an option to realize electronic devices with much higher switching speed. This is especially interesting for high frequency applications where the processed signals are much faster than the thermal system.

## Conclusion

Similar to inorganic electronics, organic transistors operating at high power reach now the level of self-heating, especially when low thermal conductive substrates are used. We demonstrate the measurement of an S-shaped NDR in a vertical organic transistor, which stems from non-linear self-heating effects. The analytical solution based on an Arrhenius-like activation can account for all aspects of the experiment. Still, this situation will add several constraints and restrictions to the circuit design in future in order to prevent thermal runaway. At the same time, the positive activation energies of the electrical conductivity are so high that even a moderate temperature increase leads to much higher performance. Thus, self-heating can become a natural way to boost the highest currents and frequencies and extend the operation and thus the application range.

## Methods

### Sample preparation

The OPBTs presented are built in a single chamber UHV-tool and on one glass substrate previously cleaned with N- Methylpyrrolidone, distilled water, ethanol, and ultra-violet ozone cleaning system. By using thermal vapor deposition at high vacuum conditions (*p* < 10^−7^ mbar), the layer stack (s. Fig. 1) is realized by subsequently depositing thin films through laser-cut, stainless steel shadow masks. The deposition system includes a wedge for realizing samples of different layer thickness in one run while other layers remain equal. The layer stack, evaporation rates and treatments of the OPBTs are: Al 100 nm (0.1 nm/s)/Cr 10 nm (0.01 nm/s)/i-C_60_ 400 nm (0.1 nm/s)/Al 15 nm (0.1 nm/s)/15 min oxidation at air/i-C_60_ 50 nm/SiO 200 nm with a free stripe of 0.2 mm (0.1 nm/s)/n-C_60_ 20 nm (0.04 nm/s) co-evaporating C_60_ with W_2_(hpp)_4_ (purchased from Novaled AG, Dresden) using 1 wt%/Cr 10 nm (0.01 nm/s)/Al 100 nm (0.1 nm/s)/encapsulation in a nitrogen atmosphere using UV cured epoxy glue without UV exposure of the active area/annealing for 2 h at 150 °C in a nitrogen glove-box on a heat plate.

### Device characterization

Electrical DC-characteristics are measured with a parameter analyzer Keithley 4200-SCS, and with a source measure unit (SMU Keithley 2602A). The thermal image is taken by an IR camera VarioTHERM head II (InfraTec GmbH, Germany) with a macro lens (JENOPTIK AG, Germany).
